# Complete genome sequence of *Thermanaerovibrio acidaminovorans* type strain (Su883^T^)

**DOI:** 10.4056/sigs.40645

**Published:** 2009-11-22

**Authors:** Mansi Chovatia, Johannes Sikorski, Maren Schröder, Alla Lapidus, Matt Nolan, Hope Tice, Tijana Glavina Del Rio, Alex Copeland, Jan-Fang Cheng, Susan Lucas, Feng Chen, David Bruce, Lynne Goodwin, Sam Pitluck, Natalia Ivanova, Konstantinos Mavromatis, Galina Ovchinnikova, Amrita Pati, Amy Chen, Krishna Palaniappan, Miriam Land, Loren Hauser, Yun-Juan Chang, Cynthia D. Jeffries, Patrick Chain, Elizabeth Saunders, John C. Detter, Thomas Brettin, Manfred Rohde, Markus Göker, Stefan Spring, Jim Bristow, Victor Markowitz, Philip Hugenholtz, Nikos C. Kyrpides, Hans-Peter Klenk, Jonathan A. Eisen

**Affiliations:** 1 DOE Joint Genome Institute, Walnut Creek, California, USA; 2 DSMZ - German Collection of Microorganisms and Cell Cultures GmbH, Braunschweig, Germany; 3 Los Alamos National Laboratory, Bioscience Division, Los Alamos, New Mexico, USA; 4 Biological Data Management and Technology Center, Lawrence Berkeley National Laboratory, Berkeley, California, USA; 5 Oak Ridge National Laboratory, Oak Ridge, Tennessee, USA; 6 Lawrence Livermore National Laboratory, Livermore, California, USA; 7 HZI – Helmholtz Centre for Infection Research, Braunschweig, Germany; 8 University of California Davis Genome Center, Davis, California, USA

**Keywords:** strictly anaerobic, amino acid fermentation, thermophile, oxidative decarboxylation, lithotrophic, co-culture with *Methanobacterium thermoautotrophicum*, *Synergistales*, *Synergistetes*

## Abstract

*Thermanaerovibrio acidaminovorans* (Guangsheng *et al.* 1997) Baena *et al.* 1999 is the type species of the genus *Thermanaerovibrio* and is of phylogenetic interest because of the very isolated location of the novel phylum *Synergistetes. T. acidaminovorans* Su883^T^ is a Gram-negative, motile, non-spore-forming bacterium isolated from an anaerobic reactor of a sugar refinery in The Netherlands. Here we describe the features of this organism, together with the complete genome sequence, and annotation. This is the first completed genome sequence from a member of the phylum *Synergistetes*. The 1,848,474 bp long single replicon genome with its 1765 protein-coding and 60 RNA genes is part of the *** G****enomic* *** E****ncyclopedia of* *** B****acteria and* *** A****rchaea * project.

## Introduction

Strain Su883^T^ (= DSM 6589 = ATCC 49978) is the type strain of the species *Thermanaerovibrio acidaminovorans*, which represents the type species of the two species containing genus *Thermanaerovibrio* [1]. Strain SU883^T^ is of particular interest because it is able to ferment quite a number of amino acids [2,3], and because its metabolism is greatly enhanced in the presence of the hydrogen scavenger *Methanobacterium thermoautotrophicum,* from which several single substrates solely hydrogen is formed as reduced fermentation product [3]. The physiological properties of the organism have been studied in detail [2,3].

Here we present a summary classification and a set of features for *T. acidaminovorans* strain SU883^T^, together with the description of the complete genome sequencing and annotation.

## Classification and features

Until now, strain SU883^T^ was the only strain known from this species. Uncultured clones with a rather high degree of 16S rRNA similarity to the sequence of strain SU883^T^ (AF071414) have been obtained from mesophilic and thermophilic bioreactors treating pharmaceutical wastewater [4] (AF280844, 97.5%; AF280820, 97.7%). The sequence similarities to environmental metagenomic libraries [5,6] were below 81%, indicating a rather poor representation of closely related strains in the analyses habitats (status July 2009).

**Figure 1:**
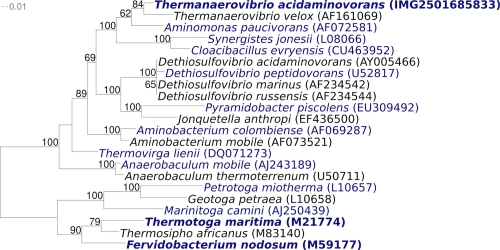
Phylogenetic tree highlighting the position of *T. acidaminovorans* strain Su883^T^ relative to the other type strains within the phylum *Synergistetes*. The tree was inferred from 1,333 aligned characters [7,8] of the 16S rRNA gene sequence under the maximum likelihood criterion [9], and was rooted with the type strains of the genera within the phylum ‘*Thermotogae*’. The branches are scaled in terms of the expected number of substitutions per site. Numbers above branches are support values from 1,000 bootstrap replicates if larger than 60%. Strains with a genome sequencing project registered in GOLD [10] are printed in blue; published genomes in bold.

Figure 1 shows the phylogenetic neighborhood of *T. acidaminovorans* strain Su883^T^ in a 16S rRNA based tree. The three 16S rRNA gene sequences in the genome of strain Su883^T^ differed from each other by up to three nucleotides, and by up to 29 nucleotides (2%) from the previously published 16S rRNA sequence, generated from DSM 6589 (AF071414). The significant difference between the genome data and the reported 16S rRNA gene sequence, which contains ten ambiguous base calls, is most likely due to sequencing errors in the previously reported sequence data.

**Figure 2:**
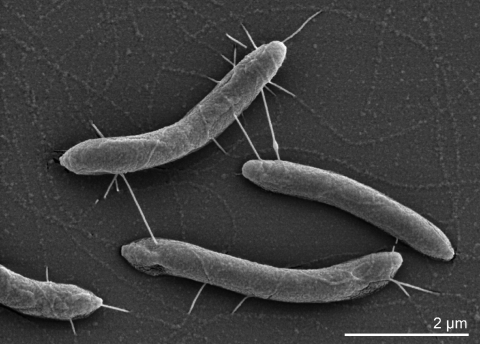
Scanning electron micrograph of *T. acidaminovorans* strain Su883^T^

*T. acidaminovorans* cells are curved rods of 0.5-0.6 × 2.5-3.0 µm in size (Table 1 and Figure 2), with round ends, occur singly, in pairs, or in long chains when grown in a complex medium [3]. The organism is Gram-negative, non-spore-forming, moderately thermophilic, motile by means of a tuft of lateral flagella at the concave side, and strictly anaerobic for growth [1]. Interestingly, it tolerates flushing with air for at least one hour, and it produces catalase [3]. While being exposed to air, strain Su883^T^ loses its motility [3]. Strain Su883^T^ is able to grow by oxidative decarboxylation of succinate to propionate. A mechanism for reductive propionate formation could be excluded [3]. Glutamate, α-ketoglutarate, histidine, arginine, ornithine, lysine, and threonine are fermented to acetate and propionate. Serine, pyruvate, alanine, glucose, fructose, xylose, glycerol and citrate are fermented to acetate. Branched-chain amino acids are converted to branched-chain fatty acids. Hydrogen is the only reduced end product [3]. The growth and the substrate conversion are strongly enhanced by co-cultivation with methanogens, e.g., *M. thermoautotrophicum* [3]. Strain Su883^T^ contains b-type cytochromes [3]. Originally, it was reported that in strain Su883^T^ thiosulfate, nitrite, sulfur and fumarate are not reduced [3]. However, a more recent study shows that, although elemental sulfur (1%) inhibits the growth of strain Su883^T^ on glucose, strain Su883^T^ could grow lithoheterotrophically with H_2_ as electron donor, S^0^ as electron acceptor, and yeast extract as carbon source [16]. The catablolism of arginine has been studied in detail. Apparently, degradation of arginine occurs by the arginine deiminase (ADI) pathway [2]. No activity of arginase, a key enzyme of the arginase pathway, could be detected [2]. No growth was observed on glycine, aspartate, gelatin, xylose, ribose, galactose, lactose, sucrose, mannose, lactate, ethanol, methanol, acetoin, betaine, malonate, and oxalate [3]. With either succinate, α-ketoglutarate or glutamate, the following enzyme activities were measured in cell free extracts: propionyl CoA:succinate IISCoA transferase, propionate kinase, acetate kinase, glutamate dehydrogenase, pyruvate dehydrogenase, α-ketoglutarate dehydrogenase, malate dehydrogenase, citrate lyase and hydrogenase [3]. The following enzymes were not detected: succinate thiokinase, fumarate reductase, succinate dehydrogenase, β-methylaspartase, hydroxyglutarate dehydrogenase, isocitrate dehydrogenase and formate dehydrogenase [3]. Unfortunately, no chemotaxonomic data are currently available for *T. acidaminovorans* strain Su883^T^.

**Table 1:** Classification and general features of *T. acidaminovorans* strain Su883^T^ according to the MIGS recommendations [11]

**MIGS ID**	**Property**	**Term**	**Evidence code**
	Current classification	Domain *Bacteria*	TAS [12]
		Phylum *Synergistetes*	TAS [13]
		Class *Synergistia*	TAS [13]
		Order *Synergistales*	TAS [13]
		Family *Synergistaceae*	TAS [13]
		Genus *Thermanaerovibrio*	TAS [1]
		Species *Thermanaerovibrio acidamonovorans*	TAS [1]
		Type strain Su883	TAS [1]
	Gram stain	negative	TAS [3]
	Cell shape	curved rods, 0.5-0.6 × 2.5-3.0 µm	TAS [3]
	Motility	motile, lateral flagella	TAS [3]
	Sporulation	non-sporulating	TAS [3]
	Temperature range	40-58°C	TAS [3]
	Optimum temperature	55°C	TAS [3]
	Salinity	no NaCl required for growth, upper tolerance border unknown	TAS [1]
MIGS-22	Oxygen requirement	strictly anaerobic	TAS [3]
	Carbon source	succinate, glucose, fructose, amongst others (see text)	TAS [3]
	Energy source	carbohydrates, amino acids	TAS [3]
MIGS-6	Habitat	granular methanogenic sludge	TAS [3]
MIGS-15	Biotic relationship	free living	NAS
MIGS-14	Pathogenicity	unknown	
	Biosafety level	1	TAS [14]
	Isolation	sludge sample taken from an upflow anaerobic sludge bed (UASB) reactor of a sugar refinery	TAS [3]
MIGS-4	Geographic location	Breda, The Netherlands	TAS [3]
MIGS-5	Sample collection time	1992 or before	TAS [3]
MIGS-4.1 MIGS-4.2	Latitude, Longitude	51.589, 4.774	NAS
MIGS-4.3	Depth	not reported	
MIGS-4.4	Altitude	not reported	

Evidence codes - IDA: Inferred from Direct Assay (first time in publication); TAS: Traceable Author Statement (i.e., a direct report exists in the literature); NAS: Non-traceable Author Statement (i.e., not directly observed for the living, isolated sample, but based on a generally accepted property for the species, or anecdotal evidence). These evidence codes are from the Gene Ontology project [15]. If the evidence code is IDA, then the property should have been directly observed for a living isolate by one of the authors, or an expert mentioned in the acknowledgements.

## Genome sequencing and annotation

### Genome project history

This organism was selected for sequencing on the basis of its phylogenetic position, and is part of the *** G****enomic* *** E****ncyclopedia of* *** B****acteria and* *** A****rchaea * project. The genome project is deposited in the Genomes OnLine Database [10] and the complete genome sequence in GenBank NOT YET. Sequencing, finishing and annotation were performed by the DOE Joint Genome Institute (JGI). A summary of the project information is shown in Table 2.

**Table 2:** Genome sequencing project information

**MIGS ID**	**Property**	**Term**
MIGS-31	Finishing quality	Finished
MIGS-28	Libraries used	Three genomic libraries: two Sanger libraries (8 kb pMCL200 and fosmid pcc1Fos) and one 454 pyrosequence standard library
MIGS-29	Sequencing platforms	ABI3730, 454 GS FLX
MIGS-31.2	Sequencing coverage	9.7x Sanger; 9.9× pyrosequence
MIGS-30	Assemblers	Newbler version 1.1.02.15, phrap
MIGS-32	Gene calling method	Prodigal, GenePRIMP
	INSDC ID	CP001818
	Genbank Date of Release	November 19, 2009
	GOLD ID	Gc01091
	INSDC project ID	29531
	Database: IMG-GEBA	2501651200
MIGS-13	Source material identifier	DSM 6589
	Project relevance	Tree of Life, GEBA

### Growth conditions and DNA isolation

*T. acidaminovorans* strain Su883^T^, DSM 6589, was grown anaerobically in DSMZ medium 104 (modified PYG medium) [17] at 55°C. DNA was isolated from 1-1.5 g of cell paste using Qiagen Genomic 500 DNA Kit (Qiagen, Hilden, Germany) following the manufacturer’s protocol without modification according to Wu *et al*. [18].

### Genome sequencing and assembly

The genome was sequenced using a combination of Sanger and 454 sequencing platforms. All general aspects of library construction and sequencing performed at the JGI can be found at the JGI website (http://www.jgi.doe.gov/). 454 Pyrosequencing reads were assembled using the Newbler assembler version 1.1.02.15 (Roche). Large Newbler contigs were broken into 2,046 overlapping fragments of 1,000 bp and 1,838 of them entered into the final assembly as pseudo-reads. The sequences were assigned quality scores based on Newbler consensus q-scores with modifications to account for overlap redundancy and to adjust inflated q-scores. A hybrid 454/Sanger assembly was made using the parallel phrap assembler (High Performance Software, LLC). Possible mis-assemblies were corrected with Dupfinisher or transposon bombing of bridging clones [19]. Gaps between contigs were closed by editing in Consed, custom primer walk or PCR amplification. A total of 401 Sanger finishing reads were produced to close gaps, to resolve repetitive regions, and to raise the quality of the finished sequence. The error rate of the completed genome sequence is less than 1 in 100,000. Together all sequence types provided 19.6 ×coverage of the genome. The final assembly contains 19,461 Sanger and 358,573 pyrosequencing reads.

### Genome annotation

Genes were identified using Prodigal [20] as part of the Oak Ridge National Laboratory genome annotation pipeline, followed by a round of manual curation using the JGI GenePRIMP pipeline (http://geneprimp.jgi-psf.org/) [21]. The predicted CDSs were translated and used to search the National Center for Biotechnology Information (NCBI) nonredundant database, UniProt, TIGRFam, Pfam, PRIAM, KEGG, COG, and InterPro databases. Additional gene prediction analysis and functional annotation was performed within the Integrated Microbial Genomes - Expert Review (http://img.jgi.doe.gov/er) platform [22].

## Genome properties

**Figure 3:**
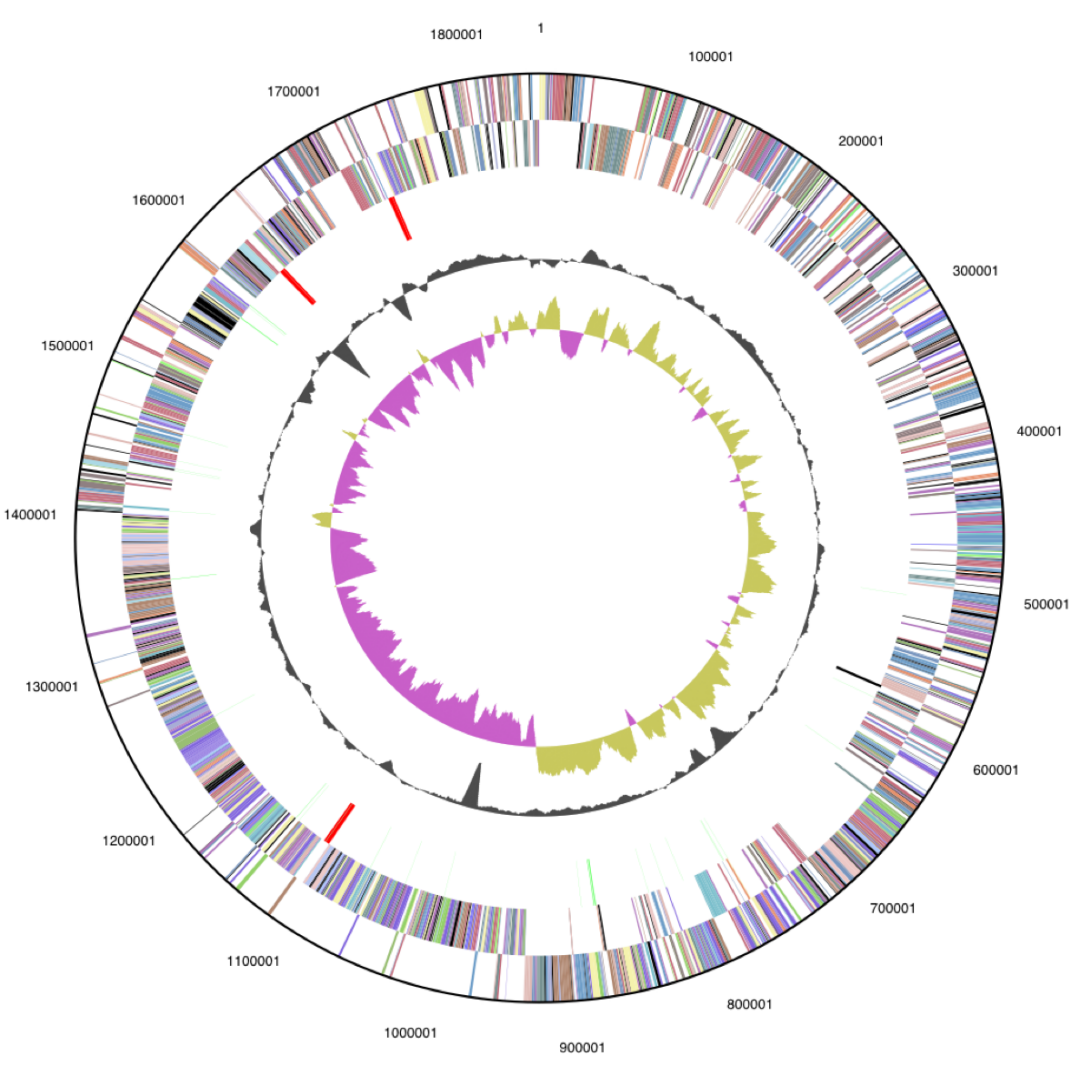
Graphical circular map of the genome. From outside to the center: Genes on forward strand (color by COG categories), Genes on reverse strand (color by COG categories), RNA genes (tRNAs green, rRNAs red, other RNAs black), GC content, GC skew.

The genome is 1,848,474 bp long and comprises one main circular chromosome with a 63.8% GC content. (Table 3, Figure 3). Of the 1,825 genes predicted, 1,765 were protein coding genes, and 60 RNAs. In addition, 27 pseudogenes were identified. The majority of genes (79.3%) were assigned a putative function while the remaining ones were annotated as hypothetical proteins. The distribution of genes into COGs functional categories is presented in Table 4.

**Table 3:** Genome Statistics

**Attribute**	**Value**	**% of Total**
Genome size (bp)	1,848,474	100.00%
DNA Coding region (bp)	1,745,505	94.43%
DNA G+C content (bp)	1,179,189	63.79%
Number of replicons	1	
Extrachromosomal elements	0	
Total genes	1,825	100.00%
RNA genes	60	3.29%
rRNA operons	3	
Protein-coding genes	1,765	96.71%
Pseudo genes	27	1.48%
Genes with function prediction	1,447	79.29%
Genes in paralog clusters	142	7.78%
Genes assigned to COGs	1,483	81.26%
Genes assigned Pfam domains	1,484	81.32%
Genes with signal peptides	275	15.07%
Genes with transmembrane helices	404	22.14%
CRISPR repeats	0	

**Table 4:** Number of genes associated with the general COG functional categories

**Code**	**Value**	**%age**	**Description**
J	150	8.5	Translation, ribosomal structure and biogenesis
A	0	0.0	RNA processing and modification
K	84	4.8	Transcription
L	71	4.0	Replication, recombination and repair
B	0	0.0	Chromatin structure and dynamics
D	26	1.5	Cell cycle control, mitosis and meiosis
Y	0	0.0	Nuclear structure
V	11	0.6	Defense mechanisms
T	101	5.7	Signal transduction mechanisms
M	97	5.5	Cell wall/membrane biogenesis
N	71	4.0	Cell motility
Z	0	0.0	Cytoskeleton
W	0	0.0	Extracellular structures
U	38	2.2	Intracellular trafficking and secretion
O	53	3.0	Posttranslational modification, protein turnover, chaperones
C	126	7.1	Energy production and conversion
G	86	4.9	Carbohydrate transport and metabolism
E	185	10.5	Amino acid transport and metabolism
F	66	3.7	Nucleotide transport and metabolism
H	97	5.5	Coenzyme transport and metabolism
I	32	1.8	Lipid transport and metabolism
P	63	3.6	Inorganic ion transport and metabolism
Q	18	1.0	Secondary metabolites biosynthesis, transport and catabolism
R	152	8.6	General function prediction only
S	104	5.9	Function unknown
-	282	16.0	Not in COGs
